# 

**Published:** 1895-05-04

**Authors:** 


**Notices of Births, Deaths, and Marriages** are inserted in this column at a charge of 2s. 6d. for an announcement not exceeding four lines.

**Notice to Advertisers and Subscribers.**

All Advertisements, Orders for Copies of The Hospital and Business Communications should be addressed to **The Manager, NOT TO THE EDITOR,** The Hospital, 428, Strand, London, W.C.

The Cost of Subscription, post free, is as follows:—Per week, 2½d.; per quarter, 2s. 8d.; per half-year, 5s. 3d.; per annum, 10s. 6d. Foreign:—Per Annum, 15s. 2d.; payable, in all cases, in advance.

### NOTICE TO CORRESPONDENTS.

All MS., Letters, Books for Review, and other matters intended for the Editor should be addressed The Editor, The Lodge, Porchester Square, London, W.

The Editor cannot undertake to return rejected MS., even when accompanied by stamped directed envelope.

**"Burdett's Hospital Annual," 1895,**

Ready Immediately.

Sixth year of publication. About 1000 pp. Scarlet cloth, gilt lettered. Price 5s. A most complete guide to British, Colonial, Indian, and American Hospitals, Dispensaries, Nursing and Convalescent Institutions, and Asylums. A few copies of the "Annual" for 1894 may still be obtained on application to the Publishers, The Scientific Press (Limited), 428, Strand, London, W.C.

**NOTICE.—Vol. XVII. of "The Hospital" (October, 1894, to April, 1895), in handsome cloth binding, is now on sale at the Office of this Journal. Price 6s. Cases for binding the Numbers contained in this Volume, 1s. 6d. Index to Vol. XVII., 2½d., post free.**

### SCRAPS AND GLEANINGS.

The Endowment Fund of the Surbiton Cottage Hospital now amounts to £1,000.

The committee of the Royal Albert Hospital, Devonport, are earnestly asking for further financial support for the institution.

The debt of £217 existent in the accounts of the Mercy Hospital, Cork, at the commencement of the year has now been reduced to £66.

Much difficulty is being experienced by the promoters of the hospital scheme at Ilfracombe in finding a site suitable for the erection of the building.

The Goldsmiths' Company has granted £500, the Vintners' Company £52 10s., and the Dyers' Company £25, to the special fund for St. Thomas' Hospital.

The usefulness of the Devon and Exeter Dental Hospital continues to be fully appreciated, 3,764 patients have availed themselves of this charity during the past year.

The opening ceremony in connection with the Children's Convalescent Home at Weston-super-Mare, a branch of the Bristol Hospital for Sick Women and Children, took place this month.

Considerable improvements have been effected at the Carmarthenshire Infirmary during the year, which, whilst involving outlay, have greatly tended to the health and comfort of the patients.

At a meeting last week of the subscribers to the Cornwall Trained Nurses' Home a resolution was passed to close the establishment; financial and other difficulties having proved insuperable.

Dr. Herman Weber, of London, has given the Royal College of Physicians a sum of money sufficient to establish a prize to be offered every two or three years for the best essay upon tuberculosis.

A sum of £500 has been received by the committee of the Bristol Eye Hospital from Mr. A. C. Bartholomew, of Reading, which is to be invested to perpetuate the memory of the late Francis Bartholomew, brother of the donor.

Much harm was caused to the Walsall Cottage Hospital in the recent great storm, and it is now being proposed to appeal not only for a renovation fund but for a sufficient sum to establish the hospital on a more extensive footing.

Considerable friction exists with regard to the selection of a site for the new buildings of the Cork Ophthalmic Hospital. Objections having been raised to all the so-far selected sites, others are now to be placed under consideration.

The Leeds Workpeople's Hospital Fund continues its excellent and energetic work. Sunday concerts in aid of its funds and outdoor Saturday afternoon entertainments are being arranged for. The annual gala will be held in Roundhay Park in August.

A cottage hospital has been presented to Brixham by the Misses Hogg. Hitherto cases of accident have been treated in a villa used as an infirmary; the present building will prove a great boon to the inhabitants. The cost has been £2,000, in addition to the furnishing.

The provision for isolation in the case of infectious diseases in Banffshire, to which we have previously referred, has again been brought under consideration by the trustees of Chalmer's Hospital, who have now come to certain conclusions, which are to be communicated to all the local authorities in the county.

The account sheet of the grand carnival held last year in connection with the funds of the Cardiff Infirmary has just been presented. This shows that, although the receipts amounted to £208, only £33 could be handed over to the institution, on account of the heavy and unforseen expenses connected with the scheme.

At the last meeting of the Macclesfield General Infirmary it was agreed that the workpeople at any factory or at any place of worship at which the sum of £2 is collected on behalf of the infirmary, will be empowered to nominate a person to represent them for the ensuing year, such person being deemed a subscriber.

The new Infectious Diseases Hospital in Tollemache Road, Birkenhead, was opened last month. The hospital is divided into an administrative block, isolation or private ward pavilion, three ward pavilions, laundry and disinfector block, mortuary, and van house. The buildings have been completed and finished at a total cost of £17,400.

At a meeting which took place last week at the Hull and Withernsea Convalescent Home, the committee expressed their thanks to Sir James Reckitt for his munificent gift to the Home of £1,000, as the nucleus of an endowment fund; this gift being in addition to Sir James Reckitt's previous liberality in giving half the initial cost of the Home.

The annual meeting of the Suffolk Convalescent Home has just been held. An amended form of medical certificate has come into force during the period under review, checking the admission of patients in an advanced stage of consumption. This accounts for the fact that 45 fewer patients were admitted during this year than in the previous one.

At the annual meeting of the Victoria Hospital for Sick Children at Hull, it was stated that owing to several special donations from friends of the institution, and £200 contributed by the Working Men's Committee, the board had been able to pay off the debit balance of over £600, and to contribute £107 out of revenue to close the building fund.

A female ambulance class has been started at the Bolton Infirmary At the recent half-yearly meeting of the institution it was stated that the infirmary accommodation is taxed to the utmost, and unless the annual income is greatly increased any enlargement of the building is out of the question. There are at present 111 beds, whereas there should be 150 to meet the requirements of the populous district.

The Standing Committee of the Meath Hospital and County Dublin Infirmary, in presenting their hundred and forty-first report, record much that is of a most satisfactory nature with regard to the general working of the institute. In connection with the above hospital a donation of £300 has been received from Miss Billing, of Ardnabel, for the purpose of founding a bed in memory of her brother, the late Robert Billing, of Ardnabel.

### THE STUDENT OF MEDICINE.

### APPOINTMENTS.

Dr. Atkinson, appointed Medical Officer for the Northern District of the Lancaster Union. A. H. Buchan, M.A., M.B., C.M., appointed House Physician to the Leith Hospital. D. Brown, B.Sc., M.D.Lond., appointed one of the Physicians to the Taunton and Somerset Hospital. A. W. Cameron, M.B., C.M., appointed House Surgeon to the Leith Hospital. Dr. Floyer, appointed Medical Officer for the Thorpe District of the Chertsey Union. L. de C. E. Harston, L.R.C.P.Edin., M.R.C.S., appointed Medical Officer for the Pirbright District of the Guildford Union. G. E. Helme, M.B., C.M., appointed House Surgeon to the Edinburgh Royal Maternity and Simpson Memorial Hospital. P. T. Hughes, M.B., C.M., appointed House Surgeon to the Edinburgh Royal Maternity and Simpson Memorial Hospital. Dr. Johnstone, appointed Medical Officer for the Clayton District of the Prestwich Union. G. C. Laing, M.B., C.M., appointed Medical Officer to Visit Outdoor Cases of the Leith Hospital. W. W. Lea-Arnold, M.D., B.S.Lond., F.R.C.S.Eng., appointed Assistant Lecturer on Obstetrics and Gynæcology to the Owens College, Manchester. S. McCoull, M.B., B.S.Durh., appointed Medical Officer for the Eastern Division of the Bywell District of the Hexham Union. C. B. Miller, L.R.C.P., M.R.C.S., L.S.A., L.M., appointed Medical Officer and Public Vaccinator for the Bonvilston District of the Cardiff Union. C. W. Milner, M.R.C.S.Eng., L.R.C.P.Lond., appointed Junior House Surgeon to the Nottingham Dispensary. J. C. Milroy, M.D.Edin., appointed Junior House Surgeon to the Royal Albert Edward Infirmary, Wigan. H. Robinson, L.R.C.P., L.R.C.S.Edin., appointed Deputy Medical Officer for the Hasland District of the Chesterfield Union. W. A. Sharpin, M.R.C.S.Eng., L.R.C.P.Lond., appointed House Surgeon to the Royal United Hospital, Bath. E. W. Skinner, M.D.Edin., appointed Coroner for the Rye District. H. S. Taylor, M.R.C.S., L.R.C.P., appointed Junior House Surgeon to the Clayton Hospital, Wakefield.

### VACANCIES.

The following vacancies are announced this week, the amount of salary in each case being given after the name of the institution:

### Resident Medical Officers.

Farringdon General Dispensary and Lying-in Charity, 17, Bartlett's Buildings, Holborn Circus.—Honorary Physician and Resident Medical Officer. Salary for the latter £100 per annum, with apartments and attendance. Applications to the Honorary Secretary by May 4th.

### THE STUDENT OF MEDICINE—*continued.*

West Riding Asylum, Wadsley, near Sheffield.—Fifth Assistant Medical Officer. Salary £100 per annum, rising £10 a year to £150, with board, &c. Applications to the Secretary by May 15th.

### Non-Resident Medical Officers.

Denbighshire Infirmary and General Dispensary, Denbigh.—Honorary Medical Officer, doubly qualified. Applications to the Chairman of the Committee of Management by May 14th.

North-Eastern Hospital for Children, Hackney Road, Shoreditch, N.E.—Physician to the Out-patients; must be Fellows or Members of the Royal College of Physicians of London. Applications to the Secretary by May 14th.

St. Thomas's Hospital Medical School.—Lecturer on Physiology. Applications to the Dean by May 25th.

### UNIVERSITIES AND COLLEGES.

### UNIVERSITY OF CAMBRIDGE.

CONGREGATIONS.—The congregations during the present term at which medical and surgical degrees will be conferred are fixed for May 9th, 16th, and 30th, June 6th and 18th.

SURGICAL EXAMINATIONS.—Professor W. Watson Cheyne, F.R.S., has been appointed an additional examiner in surgery for the present term. There are nearly eighty candidates for the surgery and obstetric division of the Third M.B. Examination.

### UNIVERSITY OF DUBLIN.

The first summer commencements in Trinity Term were held in the Theatre of Trinity College, Dublin, on Friday, April 19th, when the following degrees in the Faculty of Physic were conferred by the University Caput in the presence of the Senate:—

BACCALAUREUS IN MEDICINA, IN CHIRURGIA, ET IN ARTE OBSTETRICIA.—T. H. M. Clarke.

DOCTORES IN MEDICINA.—B. I. D'Olier, J. Elliott, W. B. Stokes, G. A. Winter, B. R. Chatterton (*in absentiâ*).

### UNIVERSITY OF EDINBURGH.

The following candidates have passed the Medical Preliminary Examination: T. H. W. Alexander, J. F. Allan, J. Anderson, J. D. Anderson, W. B. Barnes, J. H. M. Bell, L. Bruce, A. Brydon, G. B. Butt, E. Cameron, W. J. Collis, J. Cook, C. W. Crooke, J. E. Dallas, A. R. Douglas, J. Duncan, J. W. I. Dunn, W. F. Easton, W. H. Elder, E. Ewart, D. C. B. Fitzwilliams, G. Grey, M.A., P. S. Haldane, A. A. Hall, R. Hamilton, G. H. Hanna, H. Harris, D. P. Hope-Johnstone, W. Hutchison, D. S. M. Izatt, F. F. C. Jagger, E. M. G. Jayne, J. C. Kennedy, W. Landsborough, L. Lawrie, R. G. Leach, R. Liddell, C. B. M'Conaghy, A. M'Ewan, W. M'Farlane, A. F. Mackay, J. G. Mackenna, J. J. Mackenzie, T. A. Mackenzie, D. M'K. Macleod, E. Macmillan, D. M. Macrae, Elizabeth Macrory, G. P. Maitland, H. Mason, Janet A. S. Mouat, R. Murray, N. Navarra, H. C. Nixon, G. J. Ovens, G. A. Pillans, P. C. Ritchie, D. J. Roberts, L. Saunders, M. S. Scott, W. Sloss, R. Sproul, C. P. Strong, T. G. Stewart, W. L. Trafford, G. R. Turner, N. N. Wade, D. L. Wall, J. Wallace, and G. Wilson.

And the following candidates have passed in the subjects named: F. E. Belcombe, English and Mathematics; W. Brown, Latin and Mathematics; Harriet J. C. Maclaren, Latin and Mathematics; K. D. Melville, Euclid and Algebra and Latin; C. B. Paul, Mathematics; and A. W. Wilson, Latin and French.

### ROYAL COLLEGE OF SURGEONS IN IRELAND: SCHOOLS OF SURGERY.

CLASS PRIZES, WINTER SESSION, 1894.

DESCRIPTIVE ANATOMY.—Junior: W. J. Anglim, 1; H. M. Clarke and J. M. Crawford (equal), 2. Senior: F. J. Palmer, 1; and R. H. D. Pope, 2.

PRACTICAL ANATOMY.—First Year: S. R. Godkin, 1; and J. J. Huston, 2. Second Year: M. W. Faulkner, 1; and D. Hadden and J. Leventon (equal), 2. Third Year: A. S. Greene, 1; and A. McMunn, 2.

PRACTICE OF MEDICINE.—D. Kennedy, 1; and W. M. Power, 2.

THEORETICAL SURGERY.—A. I. Eades, 1; and D. Kennedy, 2.

MIDWIFERY.—G. A. Robinson, 1; and J. A. McMunn, 2.

PHYSIOLOGY.—F. J. Palmer, 1; and D. Hadden, 2.

CHEMISTRY.—W. J. Anglim, 1; and S. R. Godkin, 2.

PATHOLOGY.—G. A. Robinson, 1.

PHYSICS.—W. J. Anglim and D. A. Fitzgerald (equal), 1; and M. Gavin, 2.

The Schools opened on Monday, April 1st, for the three months' courses of Operative Surgery, Practical Chemistry, Pharmacy, Public Health, Forensic Medicine, Materia Medica, Histology, Biology, and Dissection.

